# Benefits of Animal Exposure on Veterinary Students’ Understanding of Equine Behaviour and Self-Assessed Equine Handling Skills

**DOI:** 10.3390/ani9090620

**Published:** 2019-08-28

**Authors:** Lauréline Guinnefollau, Erica K. Gee, Charlotte F. Bolwell, Elizabeth J. Norman, Chris W. Rogers

**Affiliations:** 1 School of Veterinary Science, Massey University, Palmerston North 4442, New Zealand; 2 College of Sciences, Massey University, Palmerston North 4442, New Zealand; 3 School of Agriculture and Environment, Massey University, Palmerston North 4442, New Zealand

**Keywords:** horse, behaviour, welfare, veterinary teaching, equine handling

## Abstract

**Simple Summary:**

First-year veterinary students often lack recognition of horse behavioural signals and exposure to animals. Based on self-assessments, we studied their level of knowledge of equine behaviour and their equine handling competency before starting the programme. A previous exposure to horses and/or companion animals (on their own property) seemed to confer an advantage in the interpretation of equine behaviour and self-reported equine handling competency.

**Abstract:**

Horses are one of the most dangerous animals veterinarians have to work with. For many veterinary students, their first exposure to horses occurs during practical classes. To evaluate the level of knowledge students have of equine behaviour and their equine handling competency when entering the programme, 214 veterinary students (1st and 4th year) were recruited to participate in a questionnaire. Participants were asked to choose one out of 12 terms that best represented the affective state of a horse in a picture, and to self-assess their equine handling skills. Half (n = 56/115) of the first-year students correctly interpreted the horse’s behaviour. The majority had (1) a poor understanding of equine learning mechanisms and (2) poor self-rated equine handling skills. A history of pet ownership (*p* = 0.027) and the presence of horses on their family property (*p* = 0.001) were significantly associated with a correct understanding of equine behaviour. Fourth-year students were three times more likely to accurately interpret the horse’s behaviour (*p* = 0.01) and rated their handling skills higher than first-year students (*p* = 0.006). These results suggest that previous animal experience confers a considerable advantage to interpret equine behaviour and highlight the critical importance of practical training in the veterinary programme.

## 1. Introduction

Veterinarians are 9.2 (95% CI 8.12–10.42) times more likely to suffer from a severe occupational accident compared to their colleagues in the medical profession [1]. Over a 30-year career, the average veterinarian is expected to sustain 7–8 work-related injuries [2], with this number being higher than any other professional civilian occupation, according to the British Equine Veterinary Association [3]. Additionally, 26% of Australian veterinarians affirmed that they had sustained at least one injury in the previous year [4]. The risk of injury is increased when working with large animals (e.g., horses, sheep, cows, pigs, deer, goats, alpacas, and llamas), as having a recent injury is more likely for large- and mixed- (small and large) animal veterinarians compared to small animal veterinarians [4,5]. For equine veterinarians, 67% of self-reported disease or injuries are related to work, which is higher than those reported (35%) in the European workforce [6].

In studies that have investigated veterinarians’ animal-related injuries, experience is believed to be a protective factor [7]. Recent veterinary graduates (with presumably less experience) are 1.8 times more likely to have had an injury in the past 12 months compared to graduates from previous years [4]. Inexperience (39%) is also reported by veterinary students as a factor associated with equine-related injury [8].

Horses are one of the more dangerous domestic species, being the prime or second cause of animal-related fatalities amongst the general population [9,10], with 50% of equine injuries being unrelated to riding activities [11]. In most reported cases, equine-related injuries are associated with the horse showing a stress or a fear response [8,12]. The horse is a species known for its unpredictability and innate flight response [13], which may make horses one of the most dangerous animals veterinarians have to work with. As a social prey species, it is extremely sensitive to subtle changes in body posture of its conspecifics [14]. The position and tenseness of its ears, tail, mouth, head and feet are major body parts used by horses to communicate, and handlers should have a good knowledge of these body language signals to anticipate the animal’s responses [15]. An inability to do so and to react accordingly may lead horses to become defensive and more difficult to manage [16]. Therefore, a correct understanding of equine behaviour is necessary to avoid miscommunication between the handler and the horse or inadequate timing when using learning theory. Indeed, learning theory informs the way horses learn as they respond to various signals performed by the handler and behaviours are reinforced or prevented [17]. As reported by Doherty et al. [18], the correct understanding and application of learning theory when working with horses may help to mitigate work-related injuries for veterinarians.

In the veterinary environment, a recent study suggested that the recognition of horse behavioural signals was limited for some first year students [19], which may result in both higher injury risks and animal welfare issues. Indeed, animal behaviour knowledge is one component of good handling skills. Good handling skills have been defined by Coleman and Hemsworth [20] as the “general knowledge of the animal’s need, a practical experience in the care and an ability to quickly identify changes in behaviour, health or performance, as well as addressing those appropriately”. Therefore, veterinarians who show good handling skills can minimise horses’ stress and pain, and thereby lower their reactivity levels, resulting in an animal that is easier to examine and treat [18,21,22].

In Australasia, veterinary students who have grown-up in rural environments used to outnumber those coming from urban areas, but a demographic shift has occurred in the past two decades [23]. In several countries, the majority of students who enrol in veterinary degrees nowadays come from urban environments [24,25,26]. Students’ exposure to a range of domestic animal species is therefore likely to be reduced compared to students from more rural backgrounds, and some students may never have been close to horses or production animals before [27,28]. For this reason, many veterinary students’ first exposure to large animals and horses occurs during practical classes.

Investigating first-year veterinary students’ level of animal exposure prior to starting their degree would increase the current information available on their background. Although a cluster between equine experience and accurate assessment of equine behaviour has been identified in a previous study [19], there are currently no reports on (1) the level of knowledge students have of equine behaviour and (2) their equine handling self-reported ability when entering the veterinary programme. The main objectives of the current study were therefore to provide information regarding these two points, and to evaluate what, if any, type of animal exposure confers a significant advantage to veterinary students’ knowledge of equine behaviour and self-assessed equine handling competency.

## 2. Materials and Methods

For this study, 124 first-year students and 90 fourth-year students enrolled in the Bachelor of Veterinary Science of Massey University for the 2018 year were recruited. Participation was voluntary, and students were informed that the objective of this study was to investigate their interpretation of equine behaviour. A paper-based questionnaire (available in Supplementary Materials, Figure S1) was used to gather data. 

Students were asked to provide demographic and background information (e.g., gender, type of area they spent the most time in growing up and type of large animals kept on their family property), and information regarding their general animal experience (e.g., presence of pets, previous animal-related work experience, and confidence around animals) and equine experience. The type of large animals kept on students’ family property was a multiple-choice question, whereas the presence of pets was a binary question and students were asked to indicate the species they were referring to.

General equine handling skills were self-rated by students using a 5-point scale (poor, sufficient, good, very good, and excellent). Using the same scale, they were also asked to assess their ability to perform basic handling tasks required in the first year of the degree (e.g., putting a head collar, leading, grooming, putting a rug, or lifting front and hind feet of a horse).

The last section of the questionnaire focused on students’ understanding of equine behaviour and learning theory. Participants were shown a picture of a horse (picture available in Supplementary Materials, Figure S2) and asked to circle the one term from a list of 12 that best described the horse’s behaviour (Table 1). The list of 12 terms (n = 6 positive terms, n = 6 negative terms) was adapted from the Animal Welfare Indicator’s qualitative assessment descriptors [29]. The description of each term was provided to avoid misinterpretation. Based on the picture, students were asked to identify which one or more of the horse’s body parts they used to make a decision. Following this, there were several questions aimed at determining whether students were familiar with the concepts of learning theory, including positive and negative reinforcement, and positive and negative punishment. Finally, four practical situations [30] were described and students were asked if these were examples of positive/negative reinforcement or punishment. Each student was therefore given a final score of 0% (0/4 correct answer), 25% (1/4 correct answer), 50% (2/4 correct answers), 75% (3/4 correct answers) or 100% (4/4 correct answers), related to the number of correct answers given to these four practical situations.

The questionnaire was pretested with 19 participants not enrolled in the Massey University veterinary programme and was also peer reviewed by four international experts in equine behaviour and/or veterinarians. The derived data were used to determine the correct answers to the learning theory practical situations and the horse’s behaviour in picture (Annoyed will be referred to later as the “correct term” [29]).

Results were entered into MS Excel for further analysis. The questionnaire was completed by (1) first-year veterinary students on their first day of the programme, during an introductory lecture, and (2) fourth-year veterinary students at the start of an equine practical class. Unlike 1st-year students, 4th-year students underwent equine practical classes (e.g., handling, clinical examination) and lectures (e.g., equine behaviour, safety and learning theory), as well as three weeks of equine practical placement. The project was evaluated by peer review and judged to be low risk by the Massey University Human Ethics Committee (Ethics Notification Number: 4000018923).

### Statistical Analysis

An open source sample calculator was used to assess the number of respondents required to obtain a margin of error (beta) no higher than 5%. To be within a 95% confidence interval, at an alpha risk of 5%, 138 respondents were required.

Because students referred to pets as a wide range of species, only the presence of common mammal pet species (dogs, cats, rodents, ferrets, and rabbits) was included in the analysis following demographic information and background description. Equids and other large animals were not considered to be pets.

Respondent general demographic information (gender, background, pet ownership, and the presence of large animals or horses on their family property) was analysed using pivot tables and pivot charts on Microsoft Excel to generate frequency counts and percentages. Statistical analyses were performed using R 3.5.3 (R studio). Treatment of missing values was performed using the ‘*VIM*’ package (Available online: https://cran.r-project.org/web/packages/VIM/VIM.pdf) for graphical visualisation [31] and the ‘*BaylorEdPsych’* package for Little’s test [32]. Little’s test null hypothesis states that missing values are likely to be missing completely at random (MCAR) [33]. In the case of a failure to reject the null hypothesis, a listwise deletion (complete case analysis) would be performed before further statistical analysis.

Multiple correspondence analysis (MCA) (package *‘factoextra’* Available online: https://cran.r-project.org/web/packages/factoextra/factoextra.pdf) and hierarchical cluster dendrogram (package *‘ClustOfVar’* Available online: https://cran.rproject.org/web/packages/ClustOfVar/index.html) were performed to visualise the relationships between categorical variables (e.g., background, level of equine experience, and presence of large animals on their family property). A dendrogram shows the hierarchical relationship between objects/variables, and when reading such a dendrogram, the smaller the height of the link that joins two variables, the more similar these variables are [34]. Using a “bootstrap” method, the ‘ClustOfVar’ package also provided the number of clusters for a maximum stability [35].

The threshold used for statistical significance was *p* < 0.05. Within each cluster of the dendrogram, associations between categorical variables were looked at for using binary logistic regressions to identify potentially correlated variables. In the event of a correlation, the most appropriate explanatory variable was kept for further analysis. When allowed by the data, multivariable logistic models were built with the correct/incorrect interpretation of behaviour as the dependant variable, and demographics, prior animal exposure, confidence around animals and knowledge of equine behaviour as independent variables. The package ‘*car*’ (Available online: https://cran.r-project.org/web/packages/car/car.pdf) was used to calculate variance inflation factors (VIFs) in order to identify collinearity between categorical variables used as independent variables in the multivariable logistic models [36]. After a stepwise regression method to obtain the best-fitted model based on AIC (Akaike Information Criterion) and BIC (Bayesian Information Criterion) criteria, the receiver operating characteristic (ROC) curve was calculated.

## 3. Results

### 3.1. General Demographic Information

Of the 214 eligible veterinary students, 115/124 (92%) first-year and 71/90 (79%) fourth-year students completed the questionnaire. The resulting calculated sampling error was 3% at the 95% confidence level. Response rates per question ranged from 80% to 100% (mean 98.5 ± SD 2.95). Most (75%) of the responses were not missing any information. The null hypothesis (i.e., the missing data is likely to be MCAR) was not rejected after running Little’s test (*p* = 0.18).

### 3.2. Background Description

First-year veterinary students were mostly females from an urban environment (Table 2). Most respondents grew up with a pet in their household and described themselves as confident or very confident around small animals. Less than half of the students had large animals or horses on their family property when growing up. Their confidence around large animals and horses was lower, with only 50% of 1st-year and 45% of 4th-year students rating it good, or above. While most students had had contact with horses and at least one horse riding experience prior to entering the programme, only 26% considered themselves experienced with horses.

None of the demographics of fourth-year veterinary students differed significantly from those of first-year students. Most were females, from an urban environment, grew up owning at least one pet, were confident with small/large animals and horses, had had a previous contact with horses prior entering the programme, had ridden a horse and had a low level of equine experience (Table 2).

### 3.3. Knowledge of Equine Behaviour

Respectively, first-year and fourth-year students selected nine and six out of the 12 possible terms (Table 1) describing the behaviour of the horse in the picture (Table 3 presents numbers and percentages for each selected term). The terms happy, looking for contact, and pushy were never selected. Annoyed was used by just over half of the first-year students and almost 80% of the fourth-year students to describe the horse’s behaviour (Table 3). A positive term was selected by 23% of first-year students and 4% of fourth-year students (Table 4). To help them interpret the horse’s behaviour, most first-year students chose to look at the tail, ears and hind legs (80%, 67% and 65%, respectively). Compared to first-year students, and although the percentage of students selecting each of the body parts varied, a significantly higher number of fourth-year students chose the ears and hind legs to evaluate the horse’s behaviour (p < 0.001 and p = 0.009, respectively, Table 4).

Only 35% of first-year students answered positively to the question “Have you heard the term *learning theory* before?” Although most first-year students considered that they understood the principles of positive and negative reinforcement, and negative punishment (86%, 80% and 61%, respectively), less than half of them obtained a score of 50% or higher for the four practical examples (Table 4).

Compared to first-year students, more fourth-year students selected that they were aware of the term learning theory and of the concepts of positive/negative punishment and negative reinforcement (Table 4). On a 5-point scale (final score of correct answers), students’ results with regard to the practical examples of positive/negative reinforcement/punishment were greater in fourth-year (median = 2 IQR(2–3) in first year vs. 4 IQR(3–5) in fourth year; Kruskal-Wallis test: p = 1.8 × 10^−9^).

### 3.4. Self-Assessed Equine Handling Skills

Most of the first-year students rated themselves as having poor or sufficient general equine handling skills (Table 5), and a considerable number selected that they had never put on a head collar, led, groomed, put on a rug, or lifted front and hind feet of a horse (49%: 56/115, 33%: 38/115, 44%: 51/115, 54%: 62/115, 50%: 58/115 and 54%: 62/115, respectively).

Sixty-five percent (n = 46/71) of fourth-year students rated their general equine handling skills good, very good or excellent. On a 5-point scale, students’ self-assessment of their equine handing skills was greater in fourth-year (median = 2 IQR(1–4) in first year vs. 3 IQR(2–4) in fourth year; Kruskal-Wallis test: *p* = 0.004). 

### 3.5. Influence of Animal Exposure on Equine Behaviour Knowledge and Self-Assessed Equine Handling Skills of First-Year Students

**Figure 1:**
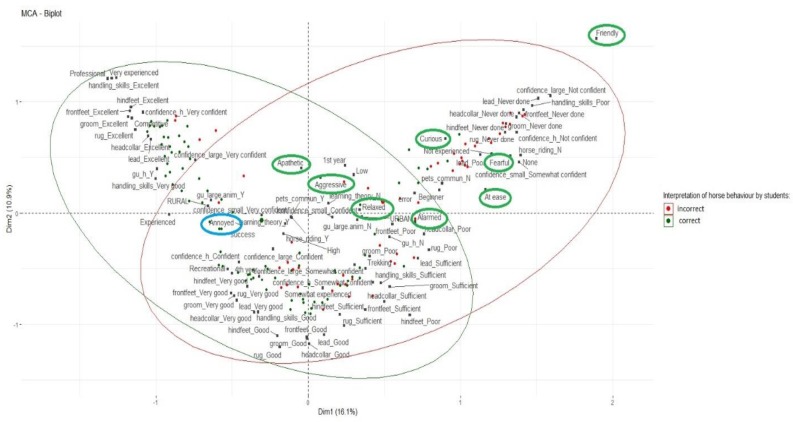
Multiple correspondence analysis (MCA) including all the variables of interest (demographics, prior animal exposure and confidence, equine experience and knowledge of equine behaviour). Circled in green, the terms selected by students to describe the horse’s behaviour; in blue, the correct term (Annoyed). The two clusters provided by the MCA represent students’ correct (in green) and incorrect (in red) interpretations of the horse’s behaviour.

An MCA including all the variables of interest showed an average profile in the middle of the plot with two clusters (Figure 1). The correct description of the horse’s behaviour was clustered with students that rated themselves as being very experienced with horses, professional and competitive riders, having excellent or very good equine handling skills, having high confidence around horses and small/large animals, and having a rural background with horses and large animals on their family property while growing up (Figure 1 shaded green). Shaded in pink in Figure 1, a selection of incorrect terms to describe the horse’s behaviour was clustered with no equine experience, a beginner horse riding level, an absence of pets, no/poor equine handling skills, and no/low confidence around small/large animals and horses.

**Figure 2:**
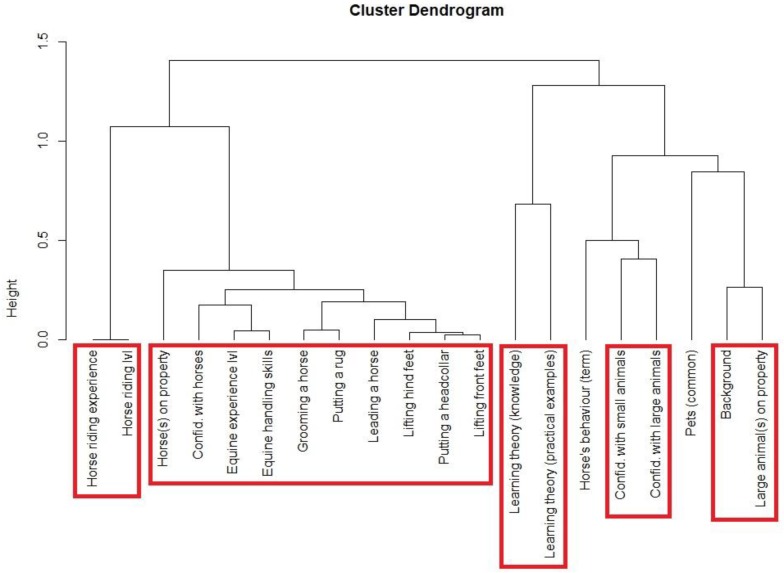
Hierarchical cluster dendrogram of the variables of interest used in the MCA. A maximum of five clusters (in red) was determined with best stability.

The hierarchical cluster dendrogram suggests several close similarities between variables (Figure 2): (1) horse riding experience and horse riding level; (2) equine handling skills, level of equine experience, confidence around horses and presence of horses on their family property; (3) knowledge of learning theory and score obtained from the practical situations; (4) confidence around small and large animals; and (5) background and presence of large animals on their family property.

Of the students that had horses on their family property when growing up, 81% (48/59) selected the correct term Annoyed. For students with no horses on their family property, 53% (63/118) described the horse as Annoyed, 15% (18/118) as Alarmed, and 11% (13/118) as Relaxed (data available in Supplementary Materials, Table S1).

All students that rated their horse riding level as “professional (e.g., instructor, trainer)” selected Annoyed. Students with lower levels of experience selected Annoyed less often: 85% for competitive or show-ring level (e.g., pony club, jumping, eventing, racing, dressage, and endurance); 77% for recreational level (e.g., hobby riding) and 56% for trekking level (e.g., organised group rides on hired horses, casual one-off paid experiences). Less than half (44%) of the beginners level (e.g., leading lesson) selected this term.

In the final statistical model including fourth-year students, three variables had a significant effect on the correct interpretation of equine behaviour: (1) the fourth year of the programme, (2) the presence of horses on their family property and (3) pet ownership; ROC = 0.65 (Table 6).

## 4. Discussion

Other than basic demographics (gender and age), very few studies have described in detail the background of veterinary students. The present study focused on gaining a better understanding of students’ knowledge of equine behaviour and self-assessed equine handling skills on entry to the undergraduate veterinary programme. To the authors’ knowledge, it is the first study to evaluate the influence, if any, of prior animal exposure on these two variables.

The overall response rate of 87% and the response rate per question suggest that the results may be representative of the whole group of veterinary students. The data of the present study agree with previous studies that report most students enrolling in undergraduate veterinary programs are females, come from an urban environment and have owned at least one pet [19,27,37,38].

Students enter the programme with various levels of knowledge of, confidence of and experience with different animal species. Similarly to Riley et al. [8], almost all students had been in contact with and ridden a horse before enrolment. However, the level of horse riding ranged from beginners to professionals and the level of equine experience from no experience to very experienced students. Moreover, the limited number of students who showed high confidence around horses suggests that a one-time horse experience might not be enough to overcome all fears when interacting with this species. The intercorrelation between confidence and time spent with horses revealed by Chamove et al. [39] supports this thinking. Therefore, significant experience may be needed to provide veterinary students with high confidence with large animals and horses found to be associated with an accurate interpretation of horse behaviour and high self-assessed equine handling skills in this study. By highlighting the importance of prior equine experience, veterinary schools could encourage students to engage early in equine-related activities. This may increase their confidence and knowledge of equine behaviour from the start of the programme and thereby mitigate the risk of injury [40]. In the present study, the horse’s ears, hind legs and tail were students’ most chosen body parts used to try assessing the horse’s behaviour, regardless of a correct or incorrect evaluation. In addition, a significant positive association was found between the selection of the ears, hind legs and tail in the decision-making and the accurate horse behaviour assessment. These are widely used parameters while assessing equine behaviour [41,42]. However, only half of the first-year students correctly interpreted the behaviour of the horse. A wide range of behaviours were selected, and 23% of students failed to recognise that the horse was in a negative affective state. Gronqvist et al. [19] found similar results, with students choosing contradictory terms to evaluate equine behaviour and being unable to assess affective states. Overall, these results suggest that most of the students had a good understanding of which of the horse’s body parts they should focus on, but many lacked competency in the way they should interpret these body signals. This suggests that prior to starting practical classes, teaching should focus on improving student’s ability to interpret the relevant body parts to identify the horses’ affective states and react accordingly with adequate cues and signals.

Indeed, the correct and well-timed use of cues and signals when interacting with a horse increases calmness and effectiveness of handling [18]. On entry to the veterinary programme, a larger proportion of veterinary students assessed themselves to have a good knowledge of the principles that constitute the way horses learn, although little more than a third (35%) had come across the term learning theory before. Similarly, their actual understanding, as measured in the questionnaire, was poor, with only 44% obtaining a score higher or equivalent to 50% when given practical examples. This falls in line with a frequent confusion between terms [43], mainly due to the use of the words “negative” and “punishment” [44]. In the context of learning theory, positive/negative stand for adding/removing a stimulus, and reinforcement/punishment for aiming to increase/decrease the frequency of a behaviour [18], as opposed to taking the words “negative” and “punishment” for their original meanings. Although horse training mainly focuses on negative reinforcement [45], even equestrian coaches are not always able to accurately articulate the learning theory terms and definitions [46]. Even though the information of this present study was obtained from a questionnaire and was not based on real-life situations, it gives a general idea of the students’ level of equine behaviour knowledge before entering the veterinary programme.

First-year students’ knowledge of equine behaviour has to be highlighted, as many veterinary schools do not include a criterion based on handling skills for selection for entry to the programme. Unsurprisingly, first-year students’ own-assessment of their competency in handling horses ranged from low (33%) to high (13%). The hierarchical cluster dendrogram suggested a close similarity between equine handling skills and level of equine experience. Although a majority of students had had at least one riding experience, the MCA also showed one cluster between a good level of horse riding and good handling skills. Similarly, there was a cluster between low level of horse riding and poor handling skills. The results provided by the MCA give a better insight of the variables clustered with an accurate interpretation of horse behaviour and good self-assessed handling competency, and start to suggest a link with previous animal experience. Similarly to the study of Gronqvist et al. [19], students’ level of experience with horses was clustered with interpretation of the horse’s behaviour.

First- and fourth-year students shared a similar demographic and background profile, which suggests a group comparison was appropriate. Towards the end of their degree, veterinary students showed a significantly greater competency of self-assessed equine handling. This result was expected, as the questions were based on techniques that students are taught during their first year of the Massey University undergraduate veterinary programme. Therefore, this may be the result of experience gained in the programme. Although large animal handling practical classes can sometimes suffer from economical and/or resources pressures [38,47], these results suggest the crucial importance of these classes in the veterinary undergraduate degrees. However, students in both years did not differ in confidence around horses, and even in fourth year, confidence around small animals was highest. This is consistent with a previous study [25], and may be due both to the unpredictability of horses and to insufficient (in quality and/or duration) external equine placements. Underlying this result could also be overconfidence among first-year students, whereas fourth-year students who have a greater understanding of the programme’s expectations in regards to their capability could have underestimated their confidence. This thinking is supported by Goldfinch et al. [48], who showed that most students entered university with a high confidence level in their skills, and revealed that previous work experience was not associated with greater confidence in first-year students’ skills.

The statistical model revealed a significant effect of the year of study on the students. This suggests that the knowledge veterinary students acquired during their degree is highly valuable for a better interpretation of equine behaviour. The presence of horses on their family property was also significant and positively associated with accurate interpretation of equine behaviour. This was expected, as most of these students were also involved in the care and management of the horses on their family property. Similarly, there was a positive association of pet ownership with an accurate interpretation of horse behaviour. While this particular result needs to be taken with caution, as most students had owned a common pet species (162/181), the necessary interactions with an animal in their household potentially prepare students for interpreting horse behaviour. This may indicate that an early exposure to common companion animal species allows students to be more attentive to horses’ subtle body and posture changes and interpret more accurately the animal’s behaviour.

Being a comparative and not a paired study is one limitation of the present work. The analysis of students’ competency in handling horses using self-assessed questions may include a bias in the form of socially desirable responding (SDR). SDR is indeed common in studies using questionnaires and investigating people’s competency, health or behaviours [49,50]. In order to limit this effect, future studies may want to independently assess students’ animal handling skills. Integrating data on equine-related students’ injuries prior to and during the veterinary programme would also bring more information in regard to students’ equine experience and handling skills.

## 5. Conclusions

Although most students had been in contact with horses before, the majority entered the veterinary programme with a low level of confidence around horses and self-assessed equine handling skills competency. Their basic understanding of equine behaviour was also low, and only half of the students correctly interpreted the horse’s behaviour. In order to keep students safe during equine practicals, these results highlight the necessity of teaching basic equine behavioural concepts and observational skills beforehand. 

Although their knowledge of equine behaviour and their self-assessed competency in handling equines increased significantly between the first- and fourth year of the programme, previous exposure to horses and/or companion animals on their family property seem to confer students a considerable advantage.

## Figures and Tables

**Table 1:** Pre-selected terms used in the questionnaire to evaluate students’ understanding of horse behaviour, adapted from the AWIN (2015).

Term	Definition
Aggressive	Hostile, defensive aggression, intention to harm
Alarmed	Worried/tense, apprehensive, nervous, on guard against a possible threat
Annoyed *	Irritated, displeased, exasperated, bothered by something, upset, troubled
Apathetic	Having or showing little or no emotion, disinterested, indifferent, unresponsive
At ease	Calm, carefree, peaceful
Curious	Inquisitive, desire to investigate
Fearful	Afraid, hesitant, timid, not confident
Friendly	Affectionate, kind, not hostile, receptive, confident
Happy	Feeling, showing or expressing joy, pleased, playful, satisfied
Looking for contact	Actively looking for interaction, interested, eager to approach
Relaxed	Not tense or rigid, easy-going, tranquil
Pushy	Assertive or forceful

* Term evaluated to be the best to describe the horse’s behaviour (referred to as “correct term”).

**Table 2:** Number and percentage of first- and fourth-year veterinary students by gender, background, confidence around animals, and equine experience. *p*-values were obtained from binary logistic regressions when comparing the distribution of variables between first- and fourth-year students.

Variables	First-Year Students Number (%)	Fourth-Year Students Number (%)	*p*-Value
Gender			
Male	21 (18%)	16 (23%)	0.497
Female	93 (82%)	55 (77%)	
Background			
Rural	40 (35%)	22 (31%)	0.582
Urban	73 (65%)	48 (69%)	
Grew up with pets (common and/or other *)	99 (89%)	65 (93%)	0.504
Large animals on their family property	52 (45%)	24 (35%)	0.189
Horses on their family property	37 (33%)	23 (32%)	0.961
Confidence (good, and above)			
With small animals	106 (92%)	66 (93%)	0.584
With large animals	58 (50%)	45 (63%)	0.681
With horses	52 (45%)	37 (52%)	0.91
General equine experience (yes)			
Previous contact with horses	106 (92%)	59 (83%)	0.063
Horse riding experience (yes/no)	102 (89%)	66 (93%)	0.344
Level of experience (good, and above)	30 (26%)	23 (32%)	0.581

* Pets (common) = dogs, cats, rabbits, rodents, ferrets. Pets (other) = fish, bird, reptiles, insects.

**Table 3:** Number and percentage of students that selected each of the 12 pre-selected terms to describe the behaviour of the horse in picture.

Term Selected	First-Year Students Number (%)	Fourth-Year Students Number (%)
Aggressive (N)	2 (2%)	2 (3%)
Alarmed (N)	13 (12%)	8 (11%)
Annoyed * (N)	56 (52%)	55 (79%)
Apathetic (N)	3 (3%)	1 (1%)
At ease (P)	5 (5%)	0
Curious (P)	5 (5%)	0
Fearful (N)	8 (7%)	1 (1%)
Friendly (P)	3 (3%)	0
Happy (P)	0	0
Looking for contact (P)	0	0
Pushy (N)	0	0
Relaxed (P)	13 (12%)	3 (4%)

* Term evaluated to be the best to describe the horse’s behaviour. N = negative term. P = positive term.

**Table 4:** Distribution of first-(n = 115/186) and fourth-year (n = 71/186) veterinary students’ responses to the evaluation of the horse’s behaviour in picture and knowledge of general equine behaviour principles. *p*-values were obtained from binary logistic regressions when comparing the distribution of variables between first- and fourth-year students.

Variables	First-Year Students Number (%)	Fourth-Year Students Number (%)	*p*-Value
Correct evaluation of horse’s behaviour	56 (52%)	55 (79%)	0.001
Positive term selected	26 (23%)	3 (4%)	0.002
Body parts used			
Back	6 (5%)	3 (4%)	0.75
Ears	76 (67%)	66 (93%)	0.0002
Eyes	36 (32%)	17 (24%)	0.27
Front legs	1 (1%)	1 (1%)	0.74
Hind legs	74 (65%)	59 (83%)	0.009
Mouth	6 (5%)	10 (14%)	0.05
Neck	21 (18%)	18 (25%)	0.26
Nostrils	8 (7%)	9 (13%)	0.2
Tail	91 (80%)	64 (90%)	0.07
Other	4 (4%)	4 (6%)	0.5
Learning theory principles–perceived knowledge of the following:			
Learning theory	39 (35%)	41 (60%)	0.001
Positive reinforcement	97 (86%)	71 (100%)	0.002
Negative reinforcement	90 (80%)	69 (98%)	0.004
Positive punishment	53 (47%)	67 (94%)	9.6 × 10^−8^
Negative punishment	68 (61%)	68 (96%)	1.5 × 10^−5^
Score ≥ 50% (practical examples)	50 (44%)	63 (89%)	4.8 × 10^−8^

**Table 5:** Number and percentage of first-(n = 115/186) and fourth-year (n = 71/186) veterinary students and their self-assessed level of equine handling skills.

Variables	First-Year Students Number (%)	Fourth-Year Students Number (%)
Self-assessed equine handling skills (good, and above)		
General equine handling skills	41 (36%)	46 (65%)
Putting a head collar on a horse	41 (36%)	60 (85%)
Leading a horse	48 (42%)	60 (86%)
Grooming a horse	41 (36%)	51 (73%)
Lifting front feet of a horse	40 (35%)	55 (77%)
Lifting hind feet of a horse	38 (33%)	46 (66%)
Putting a rug on a horse	39 (34%)	50 (71%)

**Table 6:** Best-fitted binomial logistic regression model outcomes and adjusted odds-ratios (AOR), with interpretation of equine behaviour as dependant variable, using Akaike Information Criterion (AIC). Before the stepwise regression method, the full model included the following independent variables: year of the programme, presence of pets, confidence around large animals, horse riding level, presence of horses on their family property, and knowledge of learning theory (score of practical examples).

Interpretation of Equine Behaviour (Reference = Incorrect) – AIC = 152.4	AOR	95% CI	*p*-Value
Year:			
First	Reference		
Fourth	3.02	1.33–7.17	0.01
Presence of pets:			
No	Reference		
Yes	4.89	1.3–23.78	0.027
Horses on their family property:			
No	Reference		
Yes	4.46	1.86–11.62	0.001

## Supplementary Materials

The following are available online at https://www.mdpi.com/2076-2615/9/9/620/s1, Figure S1: Questionnaire provided to first- and fourth-year veterinary students, Figure S2: Picture of the horse provided to veterinary students in the paper-based questionnaire, Table S1: Percentage of veterinary students that selected each of the 12 pre-selected terms to describe the behaviour of the horse in picture, depending on the presence or absence of horses on their family property.
