# Supplementary material for: Benefits of Animal Exposure on Veterinary Students’ Understanding of Equine Behaviour and Self-Assessed Equine Handling Skills
Source: Animals (Basel). 2019 Aug 28;9(9):620. doi: 10.3390/ani9090620 (PMC6769774; doi:10.3390/ani9090620)
Supplement: Supplementary file 1 [file animals-09-00620-s001.pdf]

animals

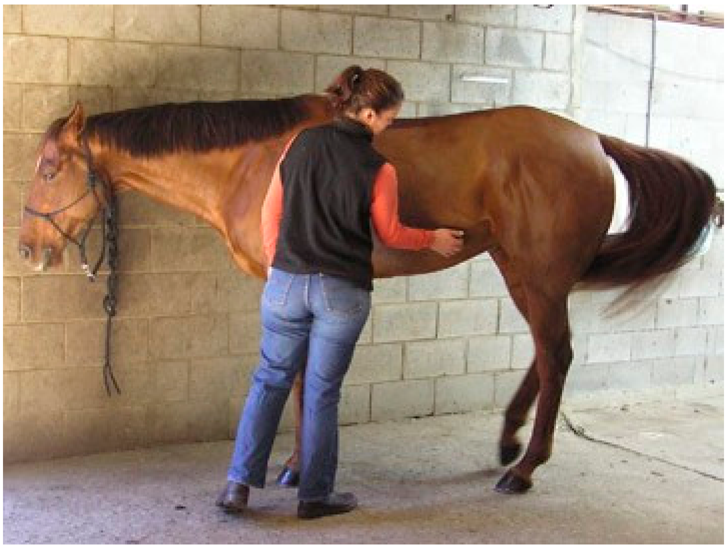

Figure S1. Picture of the horse provided to veterinary students in the paper-based questionnaire.

Term selected	Horses on property: NO	Horses on property: YES
Aggressive (N)	2.54%	1.69%
Alarmed (N)	15.25%	5.08%
Annoyed* (N)	53.39%	81.36%
Apathetic (N)	2.54%	1.69%
At ease (P)	3.39%	1.69%
Curious (P)	4.24%	0%
Fearful (N)	6.78%	1.69%
Friendly (P)	0.85%	1.69%
Happy (P)	0%	0%
Look for contact (P)	0%	0%
Pushy (N)	0%	0%
Relaxed (P)	11.02%	5.08%

* term evaluated to be the best to describe the horse's behaviour. N = negative term. P = positive term.

Table S1. Percentage of veterinary students that selected each of the 12 pre-selected terms to describe the behavior of the horse in picture, depending on the presence or absence of horses on their family property.
